# Novel NUP98:TNRC18 fusion transcript in acute myeloid leukemia: a case report and literature review

**DOI:** 10.1097/BS9.0000000000000232

**Published:** 2025-04-18

**Authors:** Lijuan Gao, Fenghong Zhang, Lijun Wen, Zheng Wang, Changgeng Ruan, Suning Chen

**Affiliations:** a National Clinical Research Center for Hematologic Diseases, Jiangsu Institute of Hematology, the First Affiliated Hospital of Soochow University, Suzhou, China; b Institute of Blood and Marrow Transplantation, Collaborative Innovation Center of Hematology, Soochow University, Suzhou, China; c Suzhou Jsuniwell Medical Laboratory, Suzhou, China

## 1. INTRODUCTION

Hematological malignancies with NUP98 rearrangement (NUP98r) were included as recurrent genetic abnormalities due to their distinctive clinicopathologic characteristics in the 2022 European LeukmiaNet (ELN) classification, the 2022 World Health Organization (WHO) classification, and the International Consensus Classification.^1–3^ NUP98r was observed in 5% to 10% of pediatric acute myeloid leukemia (AML) and 2% to 5% of adult AML, playing a role in the development and progression of AML.^4–8^ Patients with NUP98r in hematological malignancies often display a poor response to chemotherapy, a high recurrence rate, and a challenging prognosis.^9,10^

The *NUP98* gene, located on chromosome 11p15.5, encodes a 98 kDa nucleoporin, a nuclear pore complex component that is associated with cryptic translocations.^10^ The N-terminus of wild-type NUP98 is involved in the transport of RNA molecules and proteins between the cytoplasm and nucleus, as well as in the formation of biomolecular condensates, while the C-terminus plays a role in RNA binding and autoproteolytic cleavage.^11,12^ To date, the N-terminus of NUP98 has been fused to more than 40 partner genes in patients with hematological malignancies. These partner genes mainly fall into 2 categories of homeobox family genes (such as *HOXA9*, *HOXA11*, and *HOXD13*) and non-homeobox genes (such as *NSD1*, *KDM5A*, and *PAX5*).^9,13^

The *NUP98::TNRC18* fusion gene was first reported in 2022 in the largest multicenter AML cohort in China.^14^ In this case, we identified and extensively characterized a novel NUP98::TNRC18 fusion transcript in a patient with de novo AML. The discovery of this fusion gene provides further insight into the complex genetic alterations associated with AML pathogenesis.

## 2. CASE REPORT

**Figure 1.**
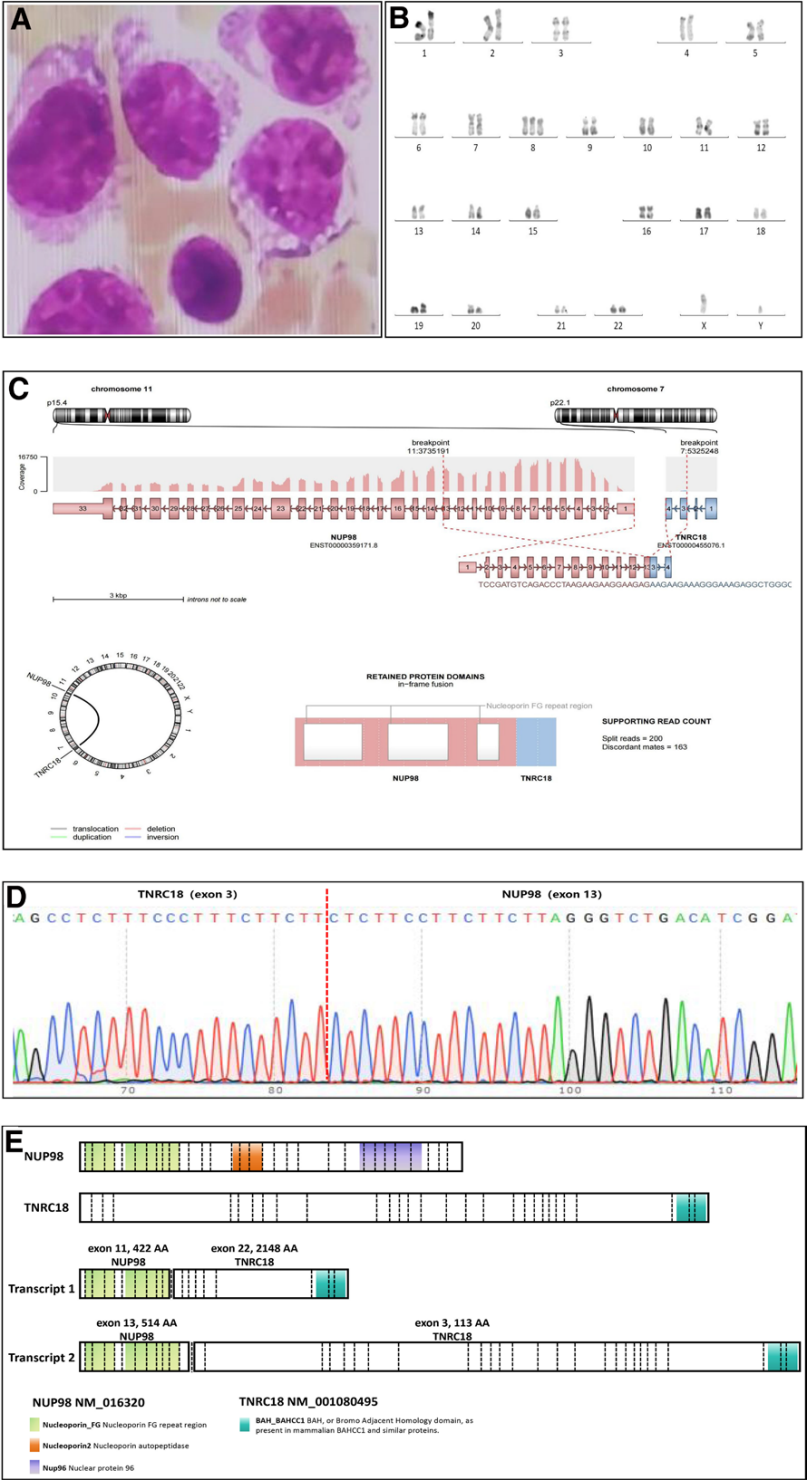
Clinical features of a patient with acute myeloid leukemia with *NUP98::TNRC18* fusion gene. (A) Bone marrow aspirate smears show blast cells. Wright-Giemsa stain, ×1000. (B) Karyotype analysis of bone marrow revealed a karyotype of 47, XY, +8 [10]. (C) RNA sequencing revealed a rare NUP98::TNRC18 fusion. The patient’s fusion transcript resulted from a translocation between exon 13 of NUP98 (located on chromosome 11) and exon 3 of TNRC18 (chromosome 7). (D) Confirmation of the breakpoint of the NUP98::TNRC18 fusion via Sanger sequencing. (E) Schematic representation of NUP98, TNRC18, and 2 transcripts of the NUP98::TNRC18 fusion. Structural domains and junction points are depicted. Transcript 1 (exon 11 of NUP98 and exon 22 of TNRC18) was previously reported.^14^ Transcript 2 (exon 13 of NUP98 and exon 3 of TNRC18) was identified in our case.

In November 2022, a 69-year-old male was admitted to our hospital, complaining of fever, fatigue, and poor appetite. A comprehensive blood cell analysis revealed a white blood cell count of 164.72 × 10^9^/L, a hemoglobin level of 132 g/L, and a platelet count of 54 × 10^9^/L. Peripheral blood aspirate smears indicated the presence of 87% blast cells. Additionally, coagulation screening showed a fibrinogen degradation product level of 25.97 μg/mL (normal range <5 μg/mL) and a D-dimer level greater than 20 mg/L (normal range: <0.55 mg/L), indicating abnormal coagulation function. Morphologic examination of bone marrow (BM) aspirate smears revealed infiltration by 80.5% blast cells (Fig. 1A). Karyotype analysis showed 47, XY, +8[10] (Fig. 1B). A multiparameter flow cytometric study on BM aspirate demonstrated that 59.9% of blast cells, which were positive for CD13, CD33, CD64, CD56, and CD38; partially positive for CD14, CD15, CD11b, CD4, CD45, and MPO; but negative for CD34, CD117, HLA-DR, CD10, CD20, CD19, CD2, CD3, CD8, CD41, and CD42b.

Cytogenetic and reverse transcription-polymerase chain reaction (RT-PCR) analyses confirmed the absence of 51 specific fusion transcripts. Additionally, no PML::RARA or BCR::ABL fusions were detected using fluorescence in situ hybridization analysis. Genetic mutation analysis of BM samples identified specific mutations, including KRAS G13D (variant allele fraction, VAF: 35.70%), TET2 S794* (VAF: 43.10%), and U2AF1 S34F (VAF: 40.40%).

To characterize the molecular aberrations, we performed targeted next-generation RNA-sequencing (RNA-seq) analysis of BM samples, which revealed a rare NUP98::TNRC18 fusion. The *NUP98* gene breakpoint occurred at chr11:3,735,191, located at the 5′ end, and the *TNRC18* gene breakpoint occurred at chr7:5,325,248, located at the 3′ end. This resulted in an in-frame *NUP98::TNRC18* fusion transcript containing exon 13 of NUP98 fused to exon 3 of TNRC18 (Fig. 1C). To further validate this fusion gene, we performed RT-PCR, Sanger sequencing, and confirmed the TNRC18::NUP98 fusion (Fig. 1D). The full-length fusion transcript is predicted to encode a 1434-amino-acid protein. Due to advanced age and poor physical condition, the patient received a low dose of cytarabine to reduce the leukocyte count. The patient died 5 days later from gastrointestinal bleeding and lung infection.

## 3. DISCUSSION

Cheng et al^14^ identified a *NUP98::TNRC18* fusion gene resulting from the fusion of exon 11 of NUP98 and exon 22 of TNRC18 in a 54-year-old male patient with AML as part of the largest multicenter AML cohort study in China. However, in this study, we identified a novel transcript of the *NUP98::TNRC18* fusion fused with exon 13 of NUP98 to exon 3 of TNRC18. A schematic representation of the 2 transcripts is shown in Figure 1E. The differences in the structures of the 2 transcripts may lead to differences in their functions. Meanwhile, we conducted a comprehensive analysis of the clinical features of both cases. Both patients were male with the same chromosomal abnormality (47, XY, +8) and rat sarcoma (RAS) mutations, consistent with the previously reported mutation spectrum of NUP98r in the literature.^9^ Both patients eventually died. Detailed information is provided in Table 1.

**Table 1:** Summary of both cases of acute myeloid leukemia with NUP98::TNRC18 fusion.

Author	Gender/age	WBC (×10^9^/L)	HGB (g/L)	PLT (×10^9^/L)	BM blast (%)	FAB type	Karyotype	Fusion location	Gene mutations (VAF)	Immunophenotype	Treatment	Outcome	Reference
Cheng et al	M/54	7.23	89	92	79.5	AML-M4	47, XY, +8/46, XY[1/25]	Exon 11 of NUP98 was fused to exon 22 of TNRC18	NRAS p.Q61H (21.7%); IDH1 p.R132C (42.4%); SMC1A p.R586Q (83%)	Positive: CD33, CD38, CD64, MPO Partially positive: CD11b, CD13, CD15, CD36, CD117, HLA-DR, CD123 Negative: CD2, CD3, CD5, CD7, CD10, CD14, CD19, CD22, CD34, CD42a, CD61, CD71, CD79a, CD138	IA	Dead	14
Present case	M/69	164.72	132	54	80.5	AML-M5	47, XY, +8[10]	Exon 13 of NUP98 was fused to exon 3 of TNRC18	KRAS p.G13D 35.70%; TET2 p.S794^*^(43.10%); U2AF1 p.S34F (40.40%)	Positive: CD13, CD33, CD64, CD56, CD38 Partially positive: CD14, CD15, CD11b, CD4, CD45, MPO Negative: CD34, CD117, HLA-DR, CD10, CD20, CD19, CD2, CD3, CD8, CD41, CD42b	Hydroxyurea, low-dose cytarabine, and leukapheresis	The patient died 5 d later due to the complications such as massive gastrointestinal bleeding	Our case

AML = acute myeloid leukemia, BM = bone marrow, FAB = French-American-British, HGB = hemoglobin, IA = idarubicin and Ara-C, M = male, PLT = platelet, VAF = variant allele fraction, WBC = white blood cell count.

Due to the cryptic nature of NUP98r, traditional cytogenetic analysis may fail to detect them. However, the advancement and widespread use of next-generation sequencing technologies, mainly RNA-Seq, have opened up new possibilities to identify novel gene fusions as molecular biomarkers for disease monitoring and treatment optimization.^15^ In this study, we used targeted RNA-Seq to identify a rare *NUP98::TNRC18* fusion in a patient with AML. This approach revealed the presence of fusion genes that were not detected using traditional methods. This highlights the power and sensitivity of RNA-seq in detecting previously unrecognized fusion events, providing valuable insights into the genomic landscape of the patient’s condition.

NUP98 fusions typically encode a fusion protein containing the N-terminal portion of NUP98 and the C-terminal portion of partner genes.^12^ To the best of our knowledge, more than 40 partner genes of NUP98 have been reported. AML with NUP98r is associated with poor prognosis, treatment failure, and high relapse rates.^16^ Numerous previous studies highlighted the crucial roles of NUP98r in the initiation and progression of AML. *NUP98* fusions have been found to disrupt normal chromatin remodeling and induce aberrant transcriptional regulation, ultimately contributing to the development of myeloid leukemogenesis. Moreover, NUP98 fusions co-occur with additional mutations, including FLT3-internal tandem duplication, which drives increased proliferation and further promotes leukemia development and progression.^17^

TNRC18 (also known as trinucleotide repeat-containing gene 18 protein), encoded by the *TNRC18* gene, which is located on chromosome 7p22.1, is predicted to have chromatin- and DNA-binding activity. Zhao et al^18^ verified that TNRC18 loss substantially activated the transcription of immunity-related genes. TNRC18::RARA fusion and XPO1::TNRC18 fusion have been reported in variant APL and AML, respectively.^19,20^ However, the specific role of TNRC18 in cancer remains unclear and requires further investigation.

In this study, we identified an in-frame NUP98::TNRC18 fusion transcript, specifically involving the fusion of exon 13 of NUP98 with exon 3 of TNRC18. The resulting fusion transcript is predicted to encode a 1434-amino-acid protein that combines the N-terminal portion of NUP98 with TNRC18. Additionally, we observed concomitant mutations in KRAS, TET2, and U2AF1, which are associated with cellular proliferation. This discovery contributes to a more comprehensive understanding of *NUP98:TNRC18* gene rearrangement in AML.

However, the mechanisms underlying leukemogenesis attributed to the NUP98::TNRC18 fusion protein remain unclear. Future mechanistic studies are warranted to gain profound insights into the role of the NUP98::TNRC18 fusion in leukemia pathogenesis. Understanding the molecular mechanisms of this fusion gene and its interactions with other genetic alterations will enhance our knowledge of AML with NUP98r and may aid in the development of targeted therapies. Nevertheless, given the poor prognosis and high relapse rate, patients with NUP98r leukemia may benefit from FLT3 inhibitors, BCL2 inhibitors, and CDK4/6 inhibitors, as well as hematopoietic stem cell transplantation.^6,21,22^

In conclusion, we identified a novel NUP98::TNRC18 fusion transcript in a patient with AML and summarized the clinical features of this disease. Both patients with only the *NUP98::TNRC18* fusion and no other fusion genes progressed rapidly and eventually died, indicating that the *NUP98::TNRC18* fusion may play a key role in the occurrence and development of leukemia. Further studies investigating the leukemogenic properties of *NUP98::TNRC18* will provide new insights into understanding this fusion.

## ACKNOWLEDGMENTS

This work was supported by grant from the National Key R&D Program of China (2019YFA0111000, 2022YFC2502700), the National Natural Science Foundation of China (82170158, 82470166, 82200149, 82100175, 82200149, 82100170), the priority academic program development of Jiangsu Higher Education Institution, the Natural Science Foundation of Jiangsu Province (BK20231195, BK20210087), the Open Project of Jiangsu Biobank of Clinical Resources (SBK202003001, SBK202003003).

All the samples were from Hematological Biobank, Jiangsu Biobank of Clinical Resources. The authors thank all the faculty and staff at the National Clinical Research Center for Hematologic Diseases, Jiangsu Institute of Hematology, the First Affiliated Hospital of Soochow University for their clinical and technical support.

## ETHICAL APPROVAL

This study was conducted in accordance with the principles of the Declaration of Helsinki and approved by the Ethics Committee of the First Affiliated Hospital of Soochow University. The ethical approval number was 2024604. Informed consent was obtained from all the patients and/or their legal guardians.

## AUTHOR CONTRIBUTIONS

L.G. wrote the manuscript. F.Z. collected the data. L.W. and Z.W. analyzed the data. C.R. and S.C. provided guidance and approved the version to be submitted. All authors contributed to the article and agreed to the published version of the manuscript.
